# The genome sequence of the Alcon Blue, *Phengaris alcon* (Denis & Schiffermüller, 1775) (Lepidoptera: Lycaenidae)

**DOI:** 10.12688/wellcomeopenres.27771.1

**Published:** 2026-09-11

**Authors:** Daniel Linke, Pável Matos-Maraví, Irena Klečková, Charlotte J. Wright, Joana I. Meier, Mark L. Blaxter, Wellcome Sanger Institute Tree of Life Management, Samples and Laboratory team, Wellcome Sanger Institute Scientific Operations: Sequencing Operations, Wellcome Sanger Institute Tree of Life Core Informatics team, Tree of Life Core Informatics collective, Project Psyche Community

**Affiliations:** 1 Biology Centre Czech Academy of Sciences, České Budějovice, South Bohemia, Czech Republic; 2 Tree of Life, Wellcome Sanger Institute, Hinxton, England, UK

**Keywords:** Phengaris alcon, Alcon Blue, genome sequence, chromosomal, Lepidoptera

## Abstract

We present a genome assembly from a female specimen of *Phengaris alcon* (Alcon Blue; Arthropoda; Insecta; Lepidoptera; Lycaenidae). The assembly contains two haplotypes with total lengths of 565.30 megabases and 536.39 megabases. Most of the haplotype 1 assembly (97.85%) was scaffolded into 23 chromosomal pseudomolecules, including the Z sex chromosome. Haplotype 2 was assembled to scaffold level. The mitochondrial genome has also been assembled, with a length of 15.22 kilobases. This work is part of Project Psyche, a collaborative programme generating genomes for European butterflies and moths.

## Species taxonomy

Eukaryota; Opisthokonta; Metazoa; Eumetazoa; Bilateria; Protostomia; Ecdysozoa; Panarthropoda; Arthropoda; Mandibulata; Pancrustacea; Altocrustacea; Allotriocarida; Hexapoda; Insecta; Dicondylia; Pterygota; Neoptera; Eumetabola; Endopterygota; Aparaglossata; Panorpida; Amphiesmenoptera; Lepidoptera; Glossata; Neolepidoptera; Heteroneura; Ditrysia; Obtectomera; Papilionoidea; Lycaenidae; Polyommatinae; *Phengaris*; *Phengaris alcon* (Denis & Schiffermüller, 1775) (NCBI:txid203773)

## Background

*Phengaris alcon*, the Alcon (Large) Blue, is a butterfly in the family Lycaenidae that occurs throughout Europe and extends into Central Asia. In Europe, populations exist from the Iberian Peninsula eastwards, reaching southern Sweden in the north, with disjunct populations in Italy and extending into the Balkans ( Tolman & Lewington, 2009). *P. alcon* is a habitat specialist with an obligate parasitic myrmecophilous larval stage. Adults have a wingspan of 25 to 40 mm, showing considerable variability in size, potentially related to overwintering for either one or two years as larvae ( Elmes *et al.*, 2019). Dorsal wings are fully blue in males and lack the dark marginal spots present in *P. teleius* or *P. nausithous*. Females have uniform brown dorsal wings with blue basal dusting, often showing dark dorsal spots. On the ventral side, *P. alcon* shows two rows of black dots, with the apical dots on the forewing distinctly displaced, which reliably separates *P. alcon* from *P. teleius*.

The taxonomy of the *P. alcon* species complex is still not fully understood. Species delimitations have been and remain contentious for *Phengaris* butterflies due to the presence of ecotypes associated with wet and dry habitats ( Koubínová *et al.*, 2017; Kudrna & Fric, 2013). The xeric (dryland) form of *P. alcon* was confused with and later merged with *P. rebeli*, described from high-altitude alpine meadows (see the review of *P. rebeli*) ( Kudrna & Fric, 2013). However, *P. rebeli* is genetically distinct and likely a separate species ( Bereczki *et al.*, 2018; Lucek *et al.*, 2024). The genetics of the *P. alcon* complex have been studied in detail (e.g. Koubínová *et al.*, 2017; Boe *et al.*, 2022; Lucek *et al.*, 2024), but pan-European genomic datasets are currently unavailable, constraining our understanding of the species complex.

The distinct ecology of xeric and hygric (wetland) *P. alcon* was first reported by Berger ( 1946), with larval foodplants differing between forms ( *Gentiana cruciata* for xeric and *G. pneumonanthe* for hygric populations). Later, genetic comparisons between ecotypes revealed repeated convergent evolution of similar habitat preferences ( Koubínová *et al.*, 2017). Adults lay their eggs on or near the flower buds of the host plants, and the larvae feed on the buds. After the second instar, caterpillars fall to the ground and are adopted by *Myrmica* ants, of which at least five species are known: *Myrmica rubra*, *M. ruginodis*, *M. scabrinodis*, *M. schencki* and *M. sabuleti* ( Als *et al.*, 2002; Sielezniew & Stankiewicz, 2007; Thomas *et al.*, 2013). Adoption is likely facilitated by rhythmic acoustic signals produced by both larvae and ant hosts ( De Gregorio *et al.*, 2026). Larvae then live as social parasites within the ant nest for either one or two winters before emerging between June and August, depending on local climatic conditions ( Elmes *et al.*, 2019). The high-altitude *P. rebeli* feeds on *Gentianella rhaetica* and parasitises *M. sulcinodis* ( Lucek *et al.*, 2024; Tartally *et al.*, 2014).

*P. alcon* is highly threatened across much of its range and listed under Appendix II of the Bern Convention and Annex IV of the EU Habitats Directive (92/43/EEC), meaning it is strictly protected across the EU and member states must ensure its habitats are maintained in favourable status. The species is now listed as Vulnerable, Endangered or even Critically Endangered in 21 of 28 European countries ( Maes *et al.* , 2019). The IUCN lists *P. alcon* as Near Threatened with decreasing populations ( van Swaay *et al.* , 2025). In Belgium, *P. alcon* has declined by 99% since 2013 and is now considered Critically Endangered, despite increased conservation action and habitat management ( Maes *et al.*, 2024). The decline is likely driven by extreme weather conditions and shifting phenology associated with climate change ( Maes *et al.*, 2024).

Conservation efforts are critical for the survival of *P. alcon* due to its dependence on the combined presence of host plant, ant host, and suitable habitat structure. Host plants ( *Gentiana*) are early-succession species and require open ground. Ant density ( *Myrmica*) must also be sufficiently high to facilitate larval adoption. One of the main threats to *P. alcon* is mowing during periods when most larvae are still present on gentian plants ( Sielezniew & Sielezniew, 2024). Climatic extremes and changes in key habitat factors (host plant and ant availability) are influencing population persistence ( WallisDeVries *et al.* , 2024). Adults are usually sedentary and do not stray far from host-plant clusters, forming colonies ( Nowicki *et al.*, 2019). Here we report the genome of an individual of the hygric form of *Phengaris alcon*, collected from a wetland in southern Bohemia (Czechia).

## Methods

### Sample acquisition

**Figure 1.**
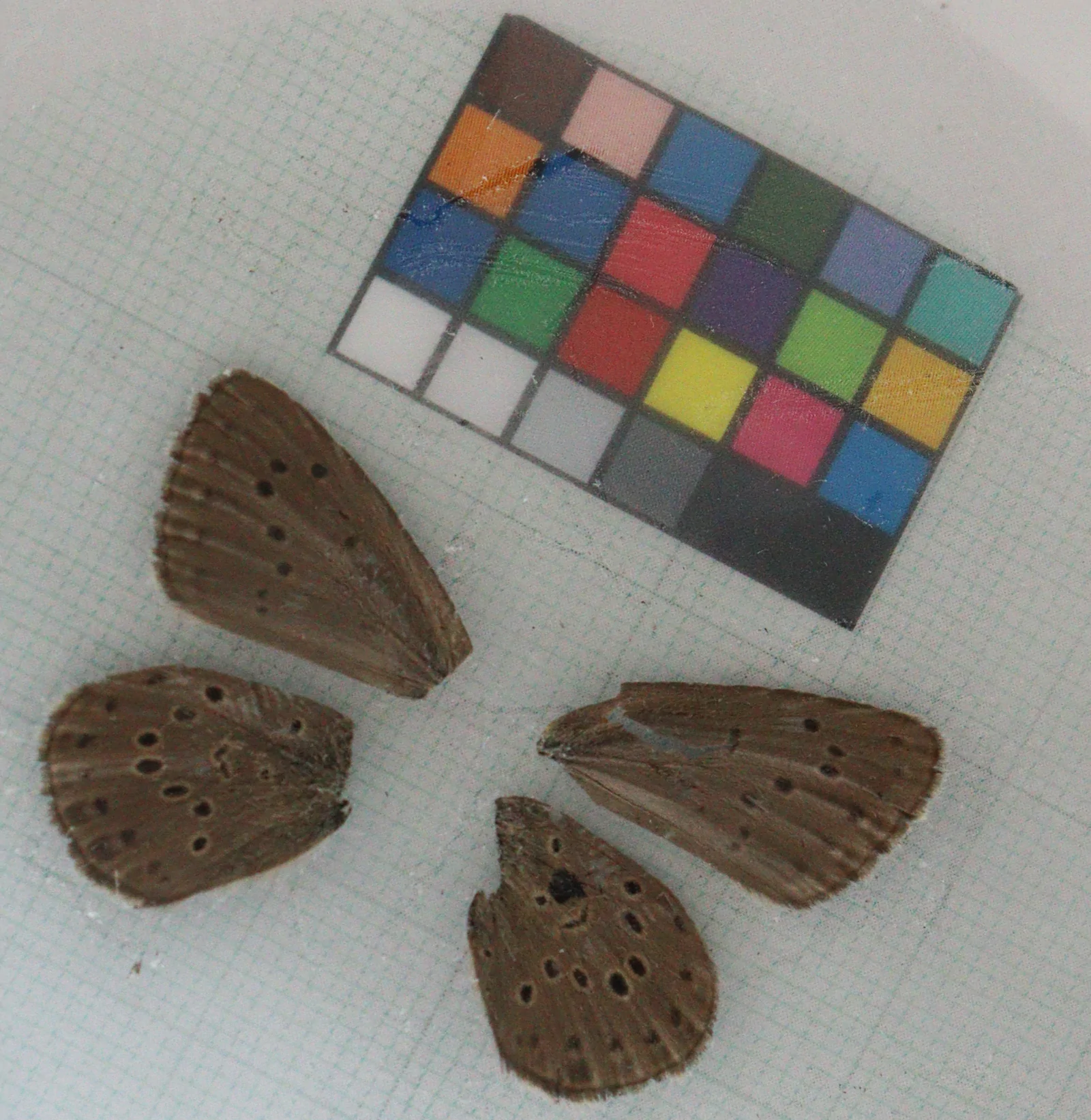
Voucher photograph of the *Phengaris alcon* (ilPheAlco1) specimen used for genome sequencing.

The specimen used for genome sequencing was an adult female *Phengaris alcon* (specimen ID BCCASPSY000073, ToLID ilPheAlco1; Figure 1), collected from Vodňany, South Bohemia, Czech Republic (latitude 49.1307, longitude 14.2548) on 2024-07-11. The specimen was collected and identified by Daniel Linke.

### Nucleic acid extraction

Protocols for high molecular weight (HMW) DNA extraction developed at the Wellcome Sanger Institute (WSI) Tree of Life Core Laboratory are available on protocols.io ( Howard *et al.*, 2025). For HMW DNA extraction, tissue from the thorax of ilPheAlco1 was homogenised by powermashing using a PowerMasher II tissue disruptor.

HMW DNA was extracted in the WSI Scientific Operations core using the Automated MagAttract v2 protocol. DNA was sheared into an average fragment size of 12–20 kb following the Megaruptor®3 for LI PacBio protocol. Sheared DNA was purified by automated SPRI (solid-phase reversible immobilisation). The concentration of the sheared and purified DNA was assessed using a Nanodrop spectrophotometer and Qubit Fluorometer using the Qubit dsDNA High Sensitivity Assay kit. Fragment size distribution was evaluated by running the sample on the Femto Pulse system. For this sample, the final post-shearing DNA had a Qubit concentration of 27.58 ng/μL and a yield of 1,296.26 ng, with a fragment size of 15.1 kb. The Genomic Quality Number (GQN) was 6.9.

### PacBio HiFi library preparation and sequencing

Library preparation and sequencing took place in the WSI Scientific Operations core. Library PSYCHE15752213 was prepared with the SMRTbell Prep Kit 3.0 (Pacific Biosciences) according to the manufacturer’s instructions. The kit includes reagents for end repair/A-tailing, adapter ligation, post-ligation SMRTbell bead clean-up, and nuclease treatment. Size selection and clean-up were performed using diluted AMPure PB beads (Pacific Biosciences). DNA concentration was quantified with a Qubit Fluorometer v4.0 (Thermo Fisher Scientific) and the Qubit 1X dsDNA HS assay kit. Final library fragment size was assessed with the Agilent Femto Pulse Automated Pulsed Field CE Instrument (Agilent Technologies) using the gDNA 55 kb BAC analysis kit.

The library was sequenced on a Revio instrument (Pacific Biosciences, California, USA). The prepared libraries selected for multiplexing were pooled based on genome size, library molarity and the intended plex level. Primers were annealed and polymerases were bound to generate circularised complexes following the manufacturer’s instructions. Complexes were purified using SMRTbell beads, diluted to the Revio loading concentration, and spiked with a Revio sequencing internal control. The pooled libraries were sequenced on a Revio 25M Plex SMRT cell in a 4-plex run. SMRT Link software (Pacific Biosciences) was used to configure and monitor the run and to perform primary and secondary data analysis.

### Hi-C

***Sample preparation and crosslinking***

Hi-C data were generated from the ilPheAlco1 head sample using the Arima-HiC v2 kit (Arima Genomics). Following the manufacturer’s instructions, tissue was fixed and DNA crosslinked using TC buffer to a final formaldehyde concentration of 2%. The tissue was homogenised using the Diagnocine Power Masher-II. Crosslinked DNA was digested with a restriction enzyme master mix, biotinylated, and ligated. Clean-up was performed with SPRISelect beads before library preparation. DNA concentration was measured with the Qubit Fluorometer (Thermo Fisher Scientific) and Qubit HS Assay Kit. The biotinylation percentage was estimated using the Arima-HiC v2 QC beads.

***Hi-C library preparation and sequencing***

Biotinylated DNA constructs were fragmented using a Covaris E220 sonicator and size-selected to 400–600 bp using SPRISelect beads. DNA was enriched with Arima-HiC v2 kit Enrichment beads. End repair, A-tailing, and adapter ligation were carried out with the NEBNext Ultra II DNA Library Prep Kit (New England Biolabs), following a modified protocol where library preparation occurs while DNA remains bound to the Enrichment beads. Libraries were amplified with KAPA HiFi HotStart mix and a custom Unique Dual Index (UDI) barcode set (Integrated DNA Technologies). Depending on sample concentration and biotinylation percentage determined at the crosslinking stage, libraries were amplified with 10–16 PCR cycles. Post-PCR clean-up was performed with SPRISelect beads. Libraries were quantified using the AccuClear Ultra High Sensitivity dsDNA Standards Assay Kit (Biotium) and a FLUOstar Omega plate reader (BMG Labtech).

Prior to sequencing, libraries were normalised to 10 ng/μL. Normalised libraries were quantified again to create equimolar and/or weighted 2.8 nM pools. Pool concentrations were checked using the Agilent 4200 TapeStation (Agilent) with High Sensitivity D500 reagents before sequencing. Libraries were sequenced on the Illumina NovaSeq X, generating paired-end 150-bp reads.

### Genome assembly

Prior to assembly of the PacBio HiFi reads, a database of *k*-mer counts ( *k* = 31) was generated from the filtered reads using FastK. The *k*-mer frequency distribution was analysed with GenomeScope (version 2.1.0) ( Ranallo-Benavidez *et al.*, 2020), providing fitted point estimates of genome size, heterozygosity and repeat content.

The HiFi reads were assembled using Hifiasm in Hi-C phasing mode ( Cheng *et al.*, 2021, 2022), producing two haplotypes. Hi-C reads ( Rao *et al.* , 2014) were mapped to contigs using bwa-mem2 ( Vasimuddin *et al.*, 2019). Contigs were further scaffolded with Hi-C data in YaHS ( Zhou *et al.*, 2023), using the --break option for handling potential misassemblies. The scaffolded assemblies were evaluated using Gfastats ( Formenti *et al.* , 2022), BUSCO ( Manni *et al.*, 2021) and MerquryFK ( Rhie *et al.*, 2020).

The mitochondrial genome was assembled using MitoHiFi ( Uliano-Silva *et al.* , 2023), which runs MitoFinder ( Allio *et al.*, 2020) and uses these annotations to select the final mitochondrial contig and to ensure the general quality of the sequence, using the mitochondrial genome of *Jamides bochus* (NC_065461.1) as the reference.

### Assembly curation

The assembly was decontaminated using the Assembly Screen for Cobionts and Contaminants ( ASCC) pipeline. The flat files and maps for curation were generated with TreeVal. Manual curation was conducted primarily in PretextView and HiGlass ( Kerpedjiev *et al.* , 2018). Scaffolds were visually inspected and corrected as described by Howe *et al*. (2021). The two haplotype assemblies were combined for curation. Manual corrections included four breaks and removal of 113 haplotypic duplications. This reduced the scaffold count by 37.4%, increased the scaffold N50 by 2.9%, and reduced the total assembly length by 0.5%. The curation process is described at sanger-tol/curation-resources. A Hi-C contact map of the haplotype 1 assembly was generated with PretextSnapshot.

### Assembly quality assessment

The MerquryFK tool ( Rhie *et al.*, 2020), run in a Singularity container ( Kurtzer *et al.*, 2017), was used to evaluate *k*-mer completeness and assembly quality for both haplotypes using the *k*-mer databases ( *k* = 31) computed prior to genome assembly. The analysis outputs included assembly QV scores and completeness statistics.

The haplotype 1 assembly was assessed using BlobToolKit ( Challis *et al.*, 2020). PacBio reads were mapped to the haplotype 1 assembly to derive sequence coverage, and BUSCO ( Manni *et al.*, 2021) was used to assess gene-set completeness. DIAMOND BLASTp searches of extracted BUSCO proteins and DIAMOND BLASTx searches of assembly sequences against UniProt Reference Proteomes ( Buchfink *et al.*, 2021), followed by BLASTn searches of sequences without DIAMOND BLASTx hits against the NCBI nt database ( Altschul *et al.*, 1990), supported taxonomic assignment. Coverage, sequence composition, taxonomic assignment and BUSCO results were integrated into a BlobDir for interactive inspection and visualisation. The snail and blob plots were rendered locally from this hosted BlobDir using BlobTk v0.8.3. The scaffold-length radial scale function for the snail plot was set to linear ( Challis & Blaxter, 2026). The blob plot used circular markers with area proportional to scaffold length ( Challis *et al.*, 2020).

We used *busco-alg-painter* v0.1.0, a Python reimplementation and extension of *lep_busco_painter*, to paint Merian elements along chromosomes. Merian elements represent the 32 ancestral linkage groups reconstructed for Lepidoptera ( Wright *et al.*, 2024). The BUSCO-to-Merian reference assignments were derived from *lep_busco_painter*. Complete single-copy and duplicated BUSCO genes from the lepidoptera_odb10 results were assigned to Merian elements. Chromosome identifiers and lengths were obtained from NCBI Datasets, and BUSCO positions were plotted on chromosomes drawn to scale.

## Genome sequence report

### Sequence data

**Figure 2.**
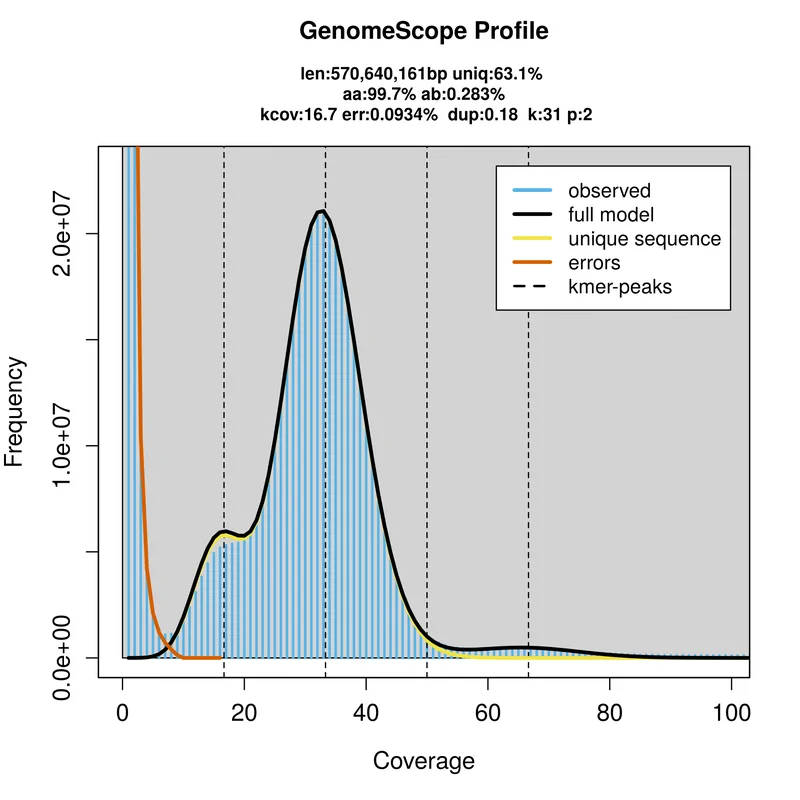
GenomeScope model fitted to the *k*-mer frequency distribution. The plot shows the observed and modelled *k*-mer spectra used to estimate genome size, heterozygosity and repeat content from unassembled sequencing reads.

PacBio sequencing of the *P. alcon* specimen generated 19.64 Gb (gigabases) from 2.18 million reads. We assembled the genome from these reads. GenomeScope analysis using a *p* = 2 model gave a *k*-mer-based haploid genome size estimate of 570.64 Mb, with heterozygosity of 0.28% and repeat content of 36.87%; the full-model fit was 98.42% (Figure 2). The GenomeScope estimates guided expectations for the assembly. The sequencing data provided approximately 33× coverage of the haplotype 1 assembly. Hi-C sequencing produced 137.26 Gb from 454.50 million reads, which were used to scaffold the haplotype 1 assembly. Table 1 summarises the specimen and sequencing details.

**Table 1.** Specimen and sequencing data for *P. alcon* (BioProject PRJEB96686)

**Platform**	**PacBio HiFi**	**Hi-C**
**ToLID**	ilPheAlco1	ilPheAlco1
**Specimen ID**	BCCASPSY000073	BCCASPSY000073
**BioSample (source individual)**	SAMEA118260579	SAMEA118260579
**BioSample (tissue)**	SAMEA118260648	SAMEA118260647
**Tissue**	thorax	head
**Instrument**	Revio (1 run)	Illumina NovaSeq X
**Library ID & run accession(s)**	PSYCHE15752213: ERR15499533	ERR15500492
**Read count total**	2.18 million reads	454.50 million read pairs
**Base count total**	19.64 Gb	137.26 Gb

### Assembly statistics

The genome was assembled into two haplotypes using Hi-C phasing. The haplotype 1 assembly was curated to chromosome level, while haplotype 2 was assembled to scaffold level. The haplotype 1 assembly, which is proposed as the reference assembly for this species, has a total length of 565.30 Mb in 111 scaffolds (excluding organelle sequences), with 65 gaps, and a scaffold N50 of 23.97 Mb (Table 2). The haplotype 1 assembly has a scaffold auN of 25.4 Mb.

**Table 2.** Genome assembly statistics for *P. alcon*

**Genome assembly**	**Haplotype 1**	**Haplotype 2**
**Assembly name**	ilPheAlco1.hap1.1	ilPheAlco1.hap2.1
**Assembly accession**	GCA_977012785.1	GCA_977012965.1
**Assembly level**	chromosome	scaffold
**Span (Mb)**	565.30	536.39
**Number of chromosomes**	23	-
**Number of contigs**	176	160
**Contig N50 (Mb)**	21.17	19.13
**Number of scaffolds (excluding organelle sequences)**	111	87
**Scaffold N50 (Mb)**	23.97	23.34
**Scaffold auN (Mb)**	25.4	25.7
**Longest scaffold length (Mb)**	46.57	48.33
**Sex chromosomes**	Z	-
**Organelles**	Mitochondrial genome: 15.22 kb	-

**Figure 3.**
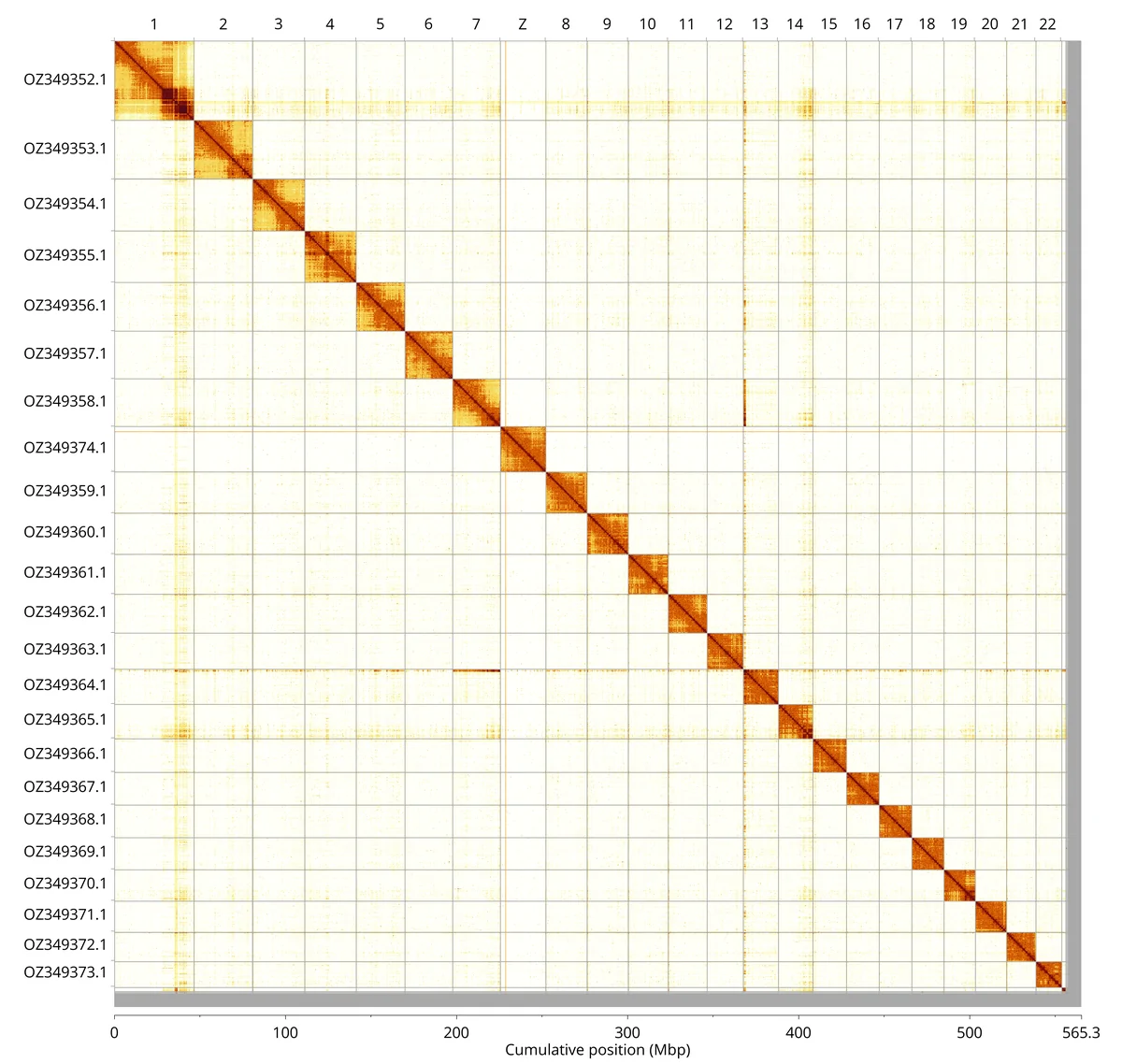
Hi-C contact map of the *P. alcon* haplotype 1 assembly. Assembled chromosomes are shown in order of size and labelled along the axes. The plot was generated using PretextSnapshot.

**Figure 4.**
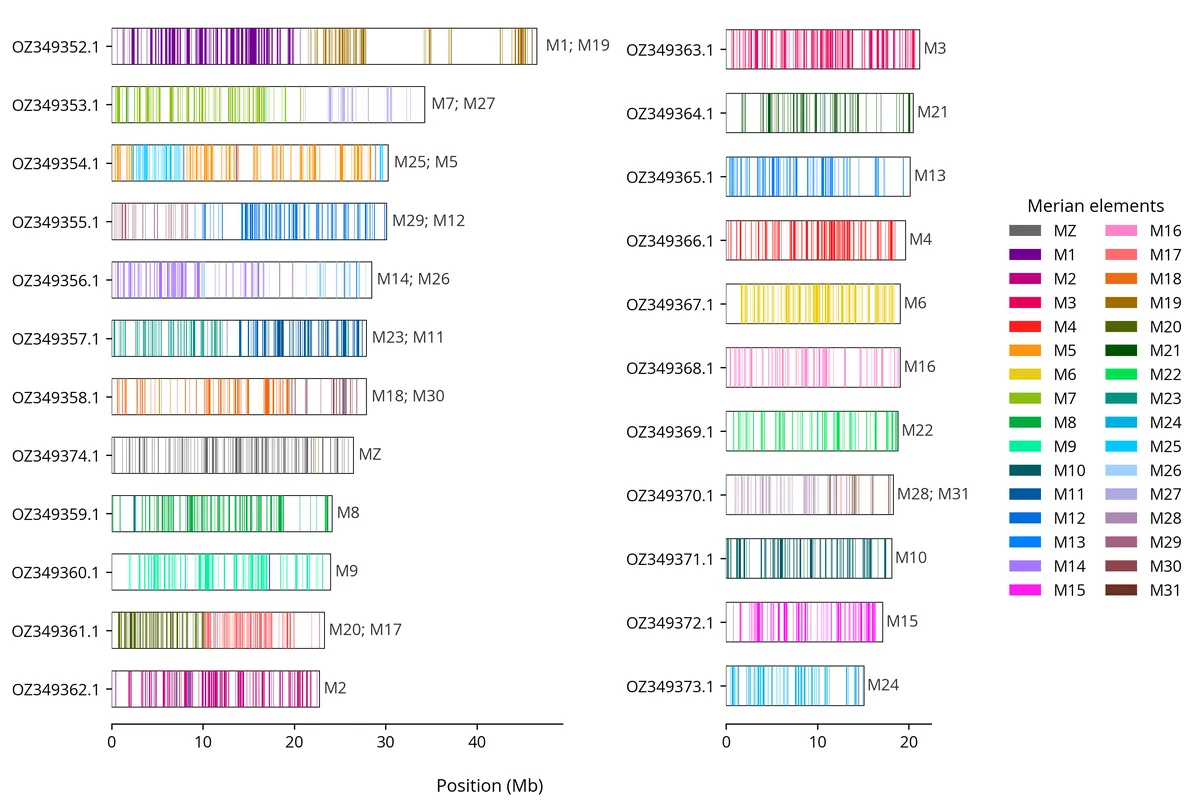
Merian elements painted across chromosomes in the ilPheAlco1.hap1.1 haplotype 1 assembly of *P. alcon*. Chromosomes are drawn to scale, with the positions of orthologues shown as coloured bars. Each orthologue is coloured by the Merian element that it belongs to. All orthologues which could be assigned to Merian elements are shown.

Most of the haplotype 1 assembly sequence (97.85%) was assigned to 23 chromosome-level scaffolds, representing 22 autosomes and the Z sex chromosome. These chromosome-level scaffolds, confirmed by Hi-C data, are named according to size (Figure 3; Table 3). Chromosome painting of the haplotype 1 assembly with Merian elements illustrates the distribution of orthologues along chromosomes and highlights patterns of chromosomal evolution relative to Lepidopteran ancestral linkage groups (Figure 4). The Z chromosome was assigned based on the presence of the ancestral Merian element MZ ( Wright *et al.*, 2024). No W chromosome was identified. The order and orientation of scaffolds in the following regions are uncertain: chromosome 1 (approximately 28.67–33.61 Mb, 35.24–36.79 Mb, and 39.83–41.95 Mb), chromosome 4 (approximately 12.52–13.68 Mb), and chromosome 13 (approximately 0–1.14 Mb).

The mitochondrial genome was also assembled (length 15.22 kb, OZ349375.1). This sequence is included as a contig in the multifasta file of the haplotype 1 genome submission and as a standalone record.

**Table 3.** Chromosomal pseudomolecules in the haplotype 1 genome assembly of *P. alcon* ilPheAlco1

**INSDC accession**	**Molecule**	**Length (Mb)**	**GC%**	**Assigned Merian elements**
OZ349352.1	1	46.57	37	M1; M19
OZ349353.1	2	34.29	37	M27; M7
OZ349354.1	3	30.29	36.50	M25; M5
OZ349355.1	4	30.09	36.50	M12; M29
OZ349356.1	5	28.46	36.50	M14; M26
OZ349357.1	6	27.91	36.50	M11; M23
OZ349358.1	7	27.87	37	M18; M30
OZ349359.1	8	24.15	36.50	M8
OZ349360.1	9	23.97	36.50	M9
OZ349361.1	10	23.29	36.50	M17; M20
OZ349362.1	11	22.74	37	M2
OZ349363.1	12	21.17	36.50	M3
OZ349364.1	13	20.48	38	M21
OZ349365.1	14	20.15	37	M13
OZ349366.1	15	19.63	37	M4
OZ349367.1	16	19.05	37	M6
OZ349368.1	17	19.05	36.50	M16
OZ349369.1	18	18.81	36.50	M22
OZ349370.1	19	18.32	37	M28; M31
OZ349371.1	20	18.13	37	M10
OZ349372.1	21	17.16	37	M15
OZ349373.1	22	15.11	37	M24
OZ349374.1	Z	26.47	36	MZ

### Assembly quality metrics

**Figure 5.**
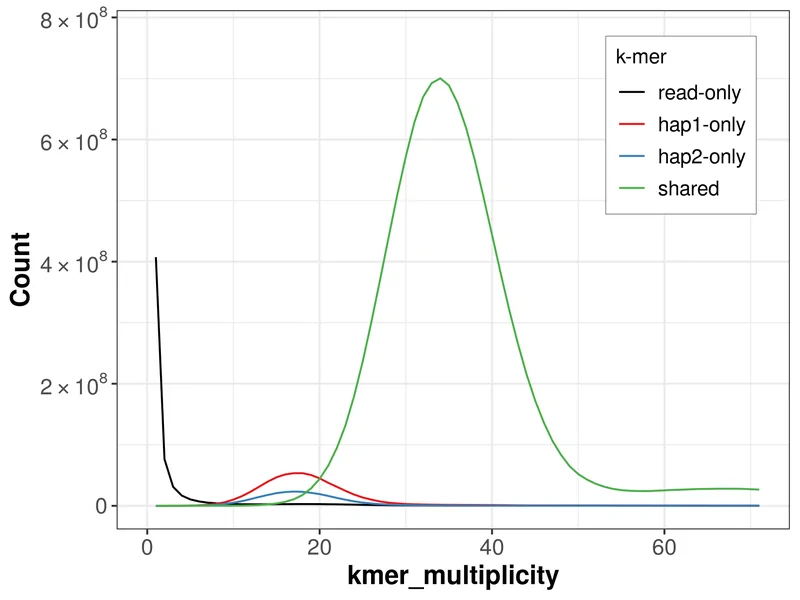
Evaluation of *k*-mer completeness using MerquryFK. This plot illustrates the recovery of *k*‐mers from the original read data in the haplotype 1 assembly, the haplotype 2 assembly and the combined haplotypes. The horizontal axis represents *k*‐mer multiplicity, and the vertical axis shows the number of *k*‐mers. The black curve represents *k*‐mers that appear in the reads but are not assembled. The green curve corresponds to *k*‐mers shared by both haplotypes, and the red and blue curves show *k*‐mers found only in one of the haplotypes.

For the haplotype 1 assembly, the estimated QV is 66.9, and for the haplotype 2 assembly, 66.9. When the two haplotypes are combined, the assembly achieves an estimated QV of 66.9. The *k*-mer completeness is 94.96% for the haplotype 1 assembly, 90.15% for the haplotype 2 assembly, and 98.71% for the combined haplotypes (Figure 5).

BUSCO analysis using the lepidoptera_odb10 reference set ( *n* = 5,286) identified 98.5% of the expected gene set (single = 98.0%, duplicated = 0.5%) in the haplotype 1 assembly. For the haplotype 2 assembly, BUSCO analysis identified 94.4% of the expected gene set (single = 93.8%, duplicated = 0.6%).

**Figure 6.**
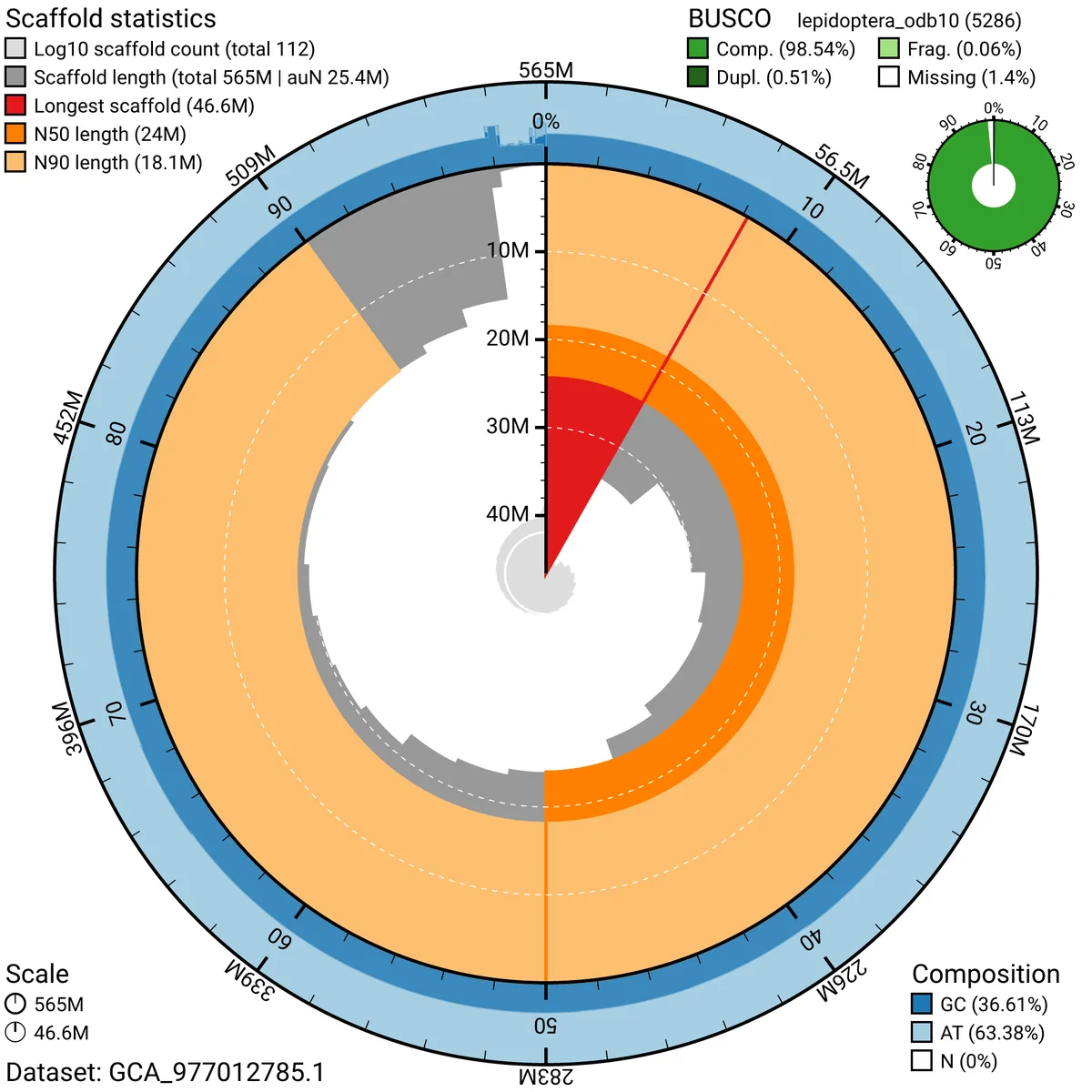
Assembly metrics for ilPheAlco1.hap1.1, the haplotype 1 assembly. The BlobToolKit snail plot provides an overview of assembly metrics and BUSCO gene completeness. The circumference represents the length of the haplotype 1 assembly, and the main plot is divided into 1,000 bins around the circumference. The outermost blue tracks display the distribution of GC, AT, and N percentages across the bins. Scaffolds are arranged clockwise from longest to shortest and are depicted in dark grey. Scaffold length is shown on a linear radial scale, scaled to the longest scaffold. Red shading indicates the longest scaffold, and the deeper orange and pale orange shading represent the N50 and N90 lengths. The summary statistics include scaffold auN, the area under the scaffold Nx curve. The light grey spiral at the centre shows the cumulative scaffold count on a logarithmic scale. A summary of complete, fragmented, duplicated, and missing BUSCO genes in the lepidoptera_odb10 set is presented at the top right. The assembly can be explored interactively in the BlobToolKit viewer.

The snail plot in Figure 6 provides a summary of assembly contiguity, cumulative scaffold count, sequence composition and BUSCO completeness for the haplotype 1 assembly ( Challis & Blaxter, 2026). The relative sizes of the longest scaffold, scaffold N50 and N90, together with the dark-grey scaffold-length distribution, show how much of the assembly is contained in long scaffolds. The outer composition track shows variation in GC, AT and N content, while the central track shows the cumulative scaffold count and the BUSCO inset summarises gene-set completeness.

**Figure 7.**
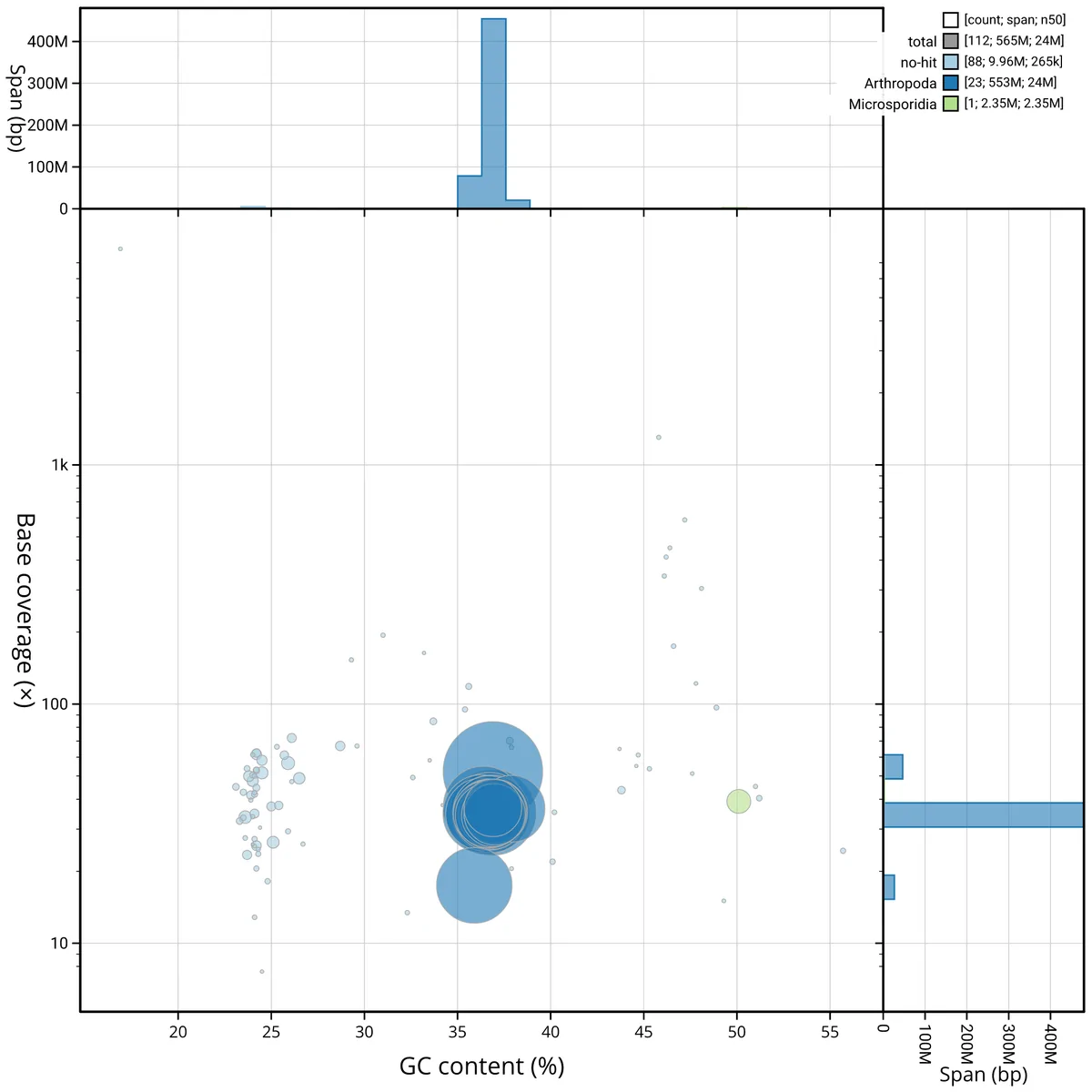
BlobToolKit blob plot for ilPheAlco1.hap1.1, the haplotype 1 assembly. The plot shows base coverage (vertical axis) and GC content (horizontal axis). Circles represent scaffolds; marker area is proportional to scaffold length, and colour indicates taxonomic assignment. The histograms along the axes display the total length of sequences distributed across different levels of coverage and GC content. The assembly can be explored interactively in the BlobToolKit viewer.

The blob plot in Figure 7 displays scaffolds from the haplotype 1 assembly by GC proportion and base coverage. Marker area is proportional to scaffold length, and colour indicates taxonomic assignment ( Challis *et al.*, 2020).

Table 4 lists the assembly metric benchmarks adapted from Rhie *et al.*. (2021) the Earth BioGenome Project (EBP) Report on Assembly Standards. The three-part EBP metric, calculated for the haplotype 1 assembly, is **7.C.Q66**.

**Table 4.** Earth BioGenome Project summary metrics for the *P. alcon* assembly

**Measure**	**Value**	***Benchmark***
EBP summary (haplotype 1)	7.C.Q66	*6.C.Q40*
Contig N50 length	21.17 Mb	*> 1 Mb*
Scaffold N50 length	23.97 Mb	*= chromosome N50*
Consensus quality (QV)	Haplotype 1: 66.9; haplotype 2: 66.9; combined: 66.9	*≥ 40*
*k*-mer completeness	Haplotype 1: 94.96%; Haplotype 2: 90.15%; combined: 98.71%	*> 90%*
BUSCO	Haplotype 1: C:98.5% [S:98.0%, D:0.5%], F:0.1%, M:1.4%, n:5,286; Haplotype 2: C:94.4% [S:93.8%, D:0.6%], F:0.1%, M:5.6%, n:5,286	*S > 90%; D < 5%*
Percentage of assembly assigned to chromosomes	97.85%	*≥ 90%*

**Notes:** The EBP summary uses log10(Contig N50); chromosome-level (C) or log10(Scaffold N50); Q (Merqury QV). BUSCO v6.0.0 using the lepidoptera_odb10 lineage dataset: C=complete; S=single-copy; D=duplicated; F=fragmented; M=missing; n=orthologues.

### Data availability

European Nucleotide Archive: Phengaris alcon. Accession number: PRJEB96686; URL: https://identifiers.org/ena.embl/PRJEB96686. The genome sequence is released openly for reuse. The *P. alcon* genome sequencing initiative is part of the Sanger Institute Tree of Life Programme ( PRJEB43745) and Project Psyche ( PRJEB71705). All raw sequence data and the haplotype assemblies have been deposited in INSDC databases. The genome will be annotated using available RNA-Seq data and presented through Ensembl at the European Bioinformatics Institute. Raw data and assembly accession identifiers are reported in Table 1 and Table 2.

Pipelines used for genome assembly at the WSI Tree of Life are available at https://pipelines.tol.sanger.ac.uk/pipelines. Table 5 lists software versions used in this study.

**Table 5.** Software versions and sources used for *P. alcon*

**Software**	**Version**	**Source**
BLAST	2.15.0+; 2.16.0+	https://ftp.ncbi.nlm.nih.gov/blast/executables/blast+/
BlobToolKit	4.4.6	https://github.com/genomehubs/blobtoolkit
BlobTk (hosted BlobDir)	0.5.1	https://github.com/genomehubs/blobtk
BlobTk (snail and blob plot renderer)	0.8.3	https://github.com/genomehubs/blobtk
BUSCO	6.0.0	https://gitlab.com/ezlab/busco
busco-alg-painter	0.1.0	https://github.com/Karenvn/busco-alg-painter
bwa-mem2	2.2.1	https://github.com/bwa-mem2/bwa-mem2
DIAMOND	2.1.8	https://github.com/bbuchfink/diamond
fasta_windows	0.2.4	https://github.com/tolkit/fasta_windows
FastK	1.2	https://github.com/thegenemyers/FASTK
GenomeScope	2.1.0	https://github.com/tbenavi1/genomescope2.0
Gfastats	1.3.6	https://github.com/vgl-hub/gfastats
Hifiasm	0.25.0-r726	https://github.com/chhylp123/hifiasm
HiGlass	1.13.4	https://github.com/higlass/higlass
MerquryFK	1.1.0-c1	https://github.com/thegenemyers/MERQURY.FK
Minimap2	2.28-r1209	https://github.com/lh3/minimap2
MitoHiFi	3.2.2	https://github.com/marcelauliano/MitoHiFi
MultiQC	1.14; 1.17 and 1.18	https://github.com/MultiQC/MultiQC
Nextflow	24.10.4	https://github.com/nextflow-io/nextflow
PretextSnapshot	0.0.6	https://github.com/sanger-tol/PretextSnapshot
PretextView	1.0.3	https://github.com/sanger-tol/PretextView
samtools	1.21	https://github.com/samtools/samtools
sanger-tol/ascc	0.1.0	https://github.com/sanger-tol/ascc
sanger-tol/blobtoolkit	v0.9.0	https://github.com/sanger-tol/blobtoolkit
sanger-tol/curationpretext	1.4.2	https://github.com/sanger-tol/curationpretext
Seqtk	1.4-r122	https://github.com/lh3/seqtk
Singularity	3.9.0	https://github.com/sylabs/singularity
TreeVal	v1.4.0	https://github.com/sanger-tol/treeval
YaHS	1.2.2	https://github.com/c-zhou/yahs

### Author information

Contributors are listed at the following links:

- Members of the Wellcome Sanger Institute Tree of Life Management, Samples and Laboratory team
- Members of Wellcome Sanger Institute Scientific Operations – Sequencing Operations
- Members of the Wellcome Sanger Institute Tree of Life Core Informatics team
- Members of the Tree of Life Core Informatics collective
- Members of the Project Psyche Community

### Wellcome Sanger Institute – Legal and Governance

The materials that have contributed to this genome note have been supplied by a Tree of Life collaborator. The Wellcome Sanger Institute employs a process whereby due diligence is carried out proportionate to the nature of the materials themselves, and the circumstances under which they have been/are to be collected and provided for use. The purpose of this is to address and mitigate any potential legal and/or ethical implications of receipt and use of the materials as part of the research project, and to ensure that in doing so, we align with best practice wherever possible. The overarching areas of consideration are:

- Ethical review of provenance and sourcing of the material
- Legality of collection, transfer and use (national and international).

Each transfer of samples is undertaken according to a Research Collaboration Agreement or Material Transfer Agreement entered into by the Tree of Life collaborator, Genome Research Limited (operating as the Wellcome Sanger Institute), and in some circumstances, other Tree of Life collaborators.
