# Supplementary material for: The genome sequence of the Alcon Blue, Phengaris alcon (Denis & Schiffermüller, 1775) (Lepidoptera: Lycaenidae)
Source: Wellcome Open Res. 2026 Sep 11;11:627. [Version 1] doi: 10.12688/wellcomeopenres.27771.1 (PMC13598397; doi:10.12688/wellcomeopenres.27771.1)
Supplement: Peer review history

# Reviewer report: Reviewer response for version 1

**DOI:** 10.21956/wellcomeopenres.30562.r169432

**Published:** 2026-09-22

**Authors:** Jinmu Kim

**Affiliations:** 1 University of Illinois at Urbana-Champaign, Urbana, Illinois, USA

**Article assessment**

 The Data Note by Linke D. et al. reports a chromosome-level genome assembly of a female *Phengaris alcon*, with a 565.3 Mb haplotype 1 assembly assigned to 22 autosomes and the Z chromosome. The reference assembly is highly contiguous, with a contig N50 of 21.17 Mb, QV 66.0. 98.5% BUSCO completeness, and 97.85% of the assembly assigned to chromosome-level. Overall, the report provides a high-quality genomic resource. However, a few minor comments should be addressed before final publication.

**Detailed comments:**

 2. Figure 1: The author should provide the size of the specimen or include a scale bar in the figure. If possible, COI sequence data could also be used to support the taxonomic identification of the specimen.

 2. The authors should clarify whether haplotype 2 was scaffolded with the same Hi-C data and explain why it remains classified as scaffolded-level assembly.

 3. Figure 3: The uncertain regions on chromosomes 1, 4, and 13 should be marked directly on the contact map using asterisks or other symbols, with details provided in the figure caption. Also, include a footnote for Table 3 about 3 chromosomes.

 4. Figure 6: The snail plot reports 112 scaffolds, whereas Table 2 reports 111 scaffolds after excluding organellar sequences. The figure caption should clarify that the snail plot includes the mitochondrial contig.

 4. Figure 7: The font size of the count labels in the figure is too small. I recommend increasing the font size to improve readability.

 5. Table 5: Explain why 3 different MultiQC versions were used in the analyses.

Are the datasets clearly presented in a usable and accessible format, and the assembly and annotation available in an appropriate subject-specific repository?

Yes

Are sufficient details of the sequencing and extraction, software used, and materials provided to allow replication by others?

Yes

Are the rationale for sequencing the genome and the species significance clearly described?

Yes

Are the protocols appropriate and is the work technically sound?

Yes

Reviewer Expertise:

Whole genome sequencing, Whole transcriptome analysis, Shotgun metagenomics, Metabarcoding, Marine genomics

I confirm that I have read this submission and believe that I have an appropriate level of expertise to confirm that it is of an acceptable scientific standard.

# Reviewer report: Reviewer response for version 1

**Published:** 2026-09-11

**Authors:** Automated Benchmarking

**Affiliations:** 1 Wellcome Sanger Institute, Hinxton, UK

As this article is a Genome Note, Automated Benchmarking has been used to determine whether the reported assembly passes three defined checks established by the Earth BioGenome Project: the raw and assembled data are publicly available, the Earth BioGenome Project three-part metric is met, and the BUSCO score passes the stated threshold. For Phengaris alcon (ilPheAlco1), all three checks passed. Data available: pass. ENA study PRJEB96686, raw-read accessions ERR15499533 and ERR15500492, and assembly accessions GCA_977012785.1 and GCA_977012965.1 are public. Earth BioGenome Project three-part metric: pass. Haplotype 1 is reported as 7.C.Q66, meeting the 6.C.Q40 reference standard. BUSCO: pass. Haplotype 1, which is proposed as the reference assembly for this species, has 98.5% complete BUSCOs (98.0% single-copy and 0.5% duplicated). It meets the target of greater than 90% complete and single-copy BUSCOs. This decision uses values reported in the Genome Note and public accession records.

Reviewer Expertise:

NA

I confirm that I have read this submission and believe that I have an appropriate level of expertise to confirm that it is of an acceptable scientific standard.
